# Current concepts and future directions in neoadjuvant chemotherapy of breast cancer

**DOI:** 10.1007/s12254-018-0421-1

**Published:** 2018-07-19

**Authors:** Rupert Bartsch, Elisabeth Bergen, Arik Galid

**Affiliations:** 1 Department of Medicine 1, Clinical Division of Oncology, Medical University of Vienna, Währinger Gürtel 18–20, 1090 Vienna, Austria; 2 Department of Gynaecology, Hanusch Hospital, Vienna, Austria

**Keywords:** Breast cancer, Neoadjuvant treatment, Chemotherapy, Biologically targeted treatment

## Abstract

Preoperative administration of chemotherapy is a widespread treatment approach in early stage breast cancer whenever chemotherapy is indicated in principle. In addition, neoadjuvant treatment is today regarded as the preferred way of delivering systemic therapy in triple-negative and HER2-positive breast cancer. While preoperative chemotherapy allows for disease downstaging and increases breast conservation rates, achieving pathologic complete remission (pCR) is usually regarded as the most pertinent aim as pCR predicts for improved long-term outcome in high-risk breast cancer subtypes. A multitude of clinical trials therefore have focused on strategies to increase pCR rates. This short review summarizes outcomes of selected studies investigating the addition of further chemotherapeutic drugs or biologically targeted agents to standard regimens and provides an overview of novel strategies currently under clinical evaluation.

## Introduction

The concept of preoperative chemotherapy was introduced nearly half a century ago in the treatment of patients with locally advanced, inoperable breast cancer [1]; this role changed over the following decades and today, neoadjuvant administration of chemotherapy is regarded as a potential standard approach whenever chemotherapy is indicated in principle [2]. Moreover, the consensus of the 2017 St. Gallen International Breast Cancer Conference defined neoadjuvant therapy as the preferred treatment approach for stage II/III triple-negative and HER2-positive breast cancer [3]. Of note, preoperative chemotherapy allows for disease downstaging and increases breast conservation rates; pathologic complete remission (pCR), however, is today regarded as the most pertinent endpoint [4].

National Surgical Breast and Bowel Project (NSABP) trial B‑27 investigated the addition of four cycles of pre- or postoperative docetaxel to four cycles of AC (doxorubicin/cyclophosphamide) [5] and neoadjuvant administration of docetaxel yielded a significant pCR improvement from 12.9% (AC) to 26.1% (AC-docetaxel). Importantly, a positive correlation of pCR with overall survival (OS) was reported as well. This observation eventually led to a multitude of studies aiming at increasing pCR rates by adding further chemotherapeutic agents, introducing new drugs instead of older ones, or adding biologically targeted agents such as antibodies or small molecules to standard treatment. In this context, the definition of pCR is relevant and today, pCR usually refers to a complete remission of invasive disease (with or without presence of ductal carcinoma in situ) in breast and axilla as this definition discriminates best between patients with favourable and unfavourable prognosis [6].

## The prognostic role of pCR

The role of pCR as surrogate for improved long-term outcome on an individual patient level in high-risk breast cancer subtypes was high-lighted by two large meta-analyses of neoadjuvant trials [6; 7]. In contrast, no clear correlation of the pCR delta with disease-free survival (DFS) or overall survival (OS) could be established on a trial level hitherto [7]. Recently, however, long-term results of the prospective randomized phase III German Breast Group (GBG) GeparSepto trial were presented [8]. In this study, substitution of conventional paclitaxel by nanoparticle-albumin bound (nab) paclitaxel resulted in a significant increase in pCR rates from 29 to 38% (odds ratio [OR] 1.53; 95% confidence interval [CI] 1.20–195; *p* = 0.00065) with the greatest effect in triple-negative breast cancer (TNBC) [9]. At a median follow-up of 49 months, this pCR difference resulted in a significant reduction of recurrence risk by absolute 6.4% (hazard ratio [HR] 0.69; 0.54–0.89). While this outcome was similar in all of the predefined subgroups, it was most pronounced in tumours with low proliferation rate (test for interaction *p* = 0.046). In TNBC, the DFS benefit in the nab-paclitaxel group was within the range predicted from the observed pCR difference; in luminal breast cancer, however, a residual effect of better chemotherapy beyond pCR must be assumed as the pCR delta was smaller in this patient subset. In summary, these data suggest that pCR most probably translates into superior outcome not only on an individual patient level but also on a trial level and is therefore a valid surrogate endpoint that has been accepted by the US Food and Drug Administration for the approval of new anti-ancer agents.

In light of the prognostic role of pCR, several studies aimed to identify biomarkers predicting for the probability of achieving pCR. Higher rates of tumour-infiltrating lymphocytes (TILs) were linked to higher pCR rates in triple-negative and HER2-positive breast cancer [10] while on the other hand, *PIK3CA* mutations predict lower pCR rates in HER2-positive disease [11]. In addition, studies investigating postneoadjuvant therapy in patients not achieving pCR in order to improve long-term outcome are ongoing. Among them, the KATHERINE study comparing adjuvant T‑DM1 to standard adjuvant trastuzumab appears to be the most promising (NCT01772472).

## Improving pCR rates

### Gemcitabine, capecitabine

pCR rates were not increased when gemcitabine was added to neoadjuvant chemotherapy in two prospective randomized phase III trials (Neo-tAnGo, NSABP B‑40) [12, 13]; a similar observation was made with the addition of capecitabine to standard chemotherapy containing anthracyclines, cyclophosphamide and sequential taxanes [11, 14]. In contrast, when capecitabine was added to six cycles of concurrent epirubicin and doxetaxel, the pCR rate was improved in the phase III ABCSG-24 trial [15]. This difference may be explained by the nonstandard cyclophosphamide-free chemotherapy backbone applied in this study and addition of gemcitabine or capecitabine is therefore not recommended when a standard regimen is considered.

### Carboplatin

The addition of weekly carboplatin AUC 1.5 (AUC2 for the first 329 patients) to weekly non-pegylated liposomal doxorubicin, paclitaxel and once every three weeks bevacizumab significantly improved pCR rate in TNBC patients in the GBG GeparSixto trial [16]. While a significant benefit in terms of DFS was reported as well [17], this prospective randomized phase II trial was criticized for its nonstandard chemotherapy backbone. Of note, the carboplatin effect was restricted to patients with *BRCA* wild-type tumours, while patients harbouring germline *BRCA* mutations had high pCR rates without an additional carboplatin benefit high-lighting the exceptional chemosensitivity of *BRCA*mut disease [17].

Significantly higher pCR rates in breast and axilla were also observed in the carboplatin cohorts of CALGB 40603, where once every three weeks carboplatin AUC6 and/or bevacizumab were added to a standard chemotherapy backbone of 12 cycles of weekly paclitaxel followed by four cycles of dose-dense AC [18]. In contrast to GeparSixto, however, no DFS benefit was reported [19]. Of note, neither of these phase II trials was powered to detect a DFS benefit and due to the size and design of the studies, no final conclusion regarding the role of carboplatin in TNBC could be drawn.

Therefore, data from the placebo-controlled phase III BrighTNess study evaluating the addition of carboplatin (with or without the PARP-inhibitor veliparib) to a standard neoadjuvant chemotherapy backbone consisting of paclitaxel weekly × 12 followed by four cycles of AC once every two or three weeks were eagerly awaited [20]. Similar to the aforementioned trials, toxicity was significantly increased in the carboplatin-containing arms; still, pCR rates were significantly higher as well (arm A 53.2% [carboplatin plus veliparib]; arm B 57.5% [carboplatin]; arm C 31% [control]; arm A *vs*. C *p* < 0.001; post hoc analysis arm B *vs*. C *p* < 0.001). While addition of veliparib to chemotherapy was therefore unsuccessful in an unselected population of TNBC patients, carboplatin increased pCR rates to a clinically relevant extent. While the optimal dose and schedule of carboplatin remains a matter of debate, adding carboplatin to standard neoadjuvant chemotherapy may now be regarded as potential standard-of-care in TNBC patients deemed as candidates for intensified treatment.

### nab-Paclitaxel

As outlined above, the GeparSepto trial evaluated the substitution of conventional paclitaxel by nab-paclitaxel. While the initial schedule of 150 mg/m^2^ weekly for 12 consecutive weeks had to be amended to 125 mg/m^2^/week due to toxicity, a significantly higher pCR rate was observed in the nab-paclitaxel group [9]. In contrast, the ETNA trial could not establish superiority of nab-paclitaxel over conventional paclitaxel [21]. ETNA, however, utilized a taxane schedule of three weeks on/one week off similar to the metastatic setting, resulting in decreased dose intensity. While not licensed for neoadjuvant treatment, preoperative therapy incorporating 12 cycles of weekly nab-paclitaxel may therefore be an option in selected patients.

### Targeted therapies

A large number of clinical trials investigated the addition of biologically targeted agents to neoadjuvant chemotherapy with varying degrees of success. To date, this strategy was most successful in HER2-positive BC, where the addition of trastuzumab, a humanized monoclonal antibody targeting HER2, to standard neoadjuvant chemotherapy more than doubled pCR rates [22]. A further increase in pCR rates was observed with dual HER2 blockade consisting of trastuzumab and pertuzumab, an antibody inhibiting HER2/HER3 heterodimerization, despite a reduction in the number of chemotherapy cycles (NEOSPHERE; TRYPHAENA) [23, 24]. The NEOSPHERE trial failed to identify a significant DFS benefit with dual-HER2 inhibition [25] but again, this phase II study was not powered to detect long-term outcome improvements. Today, six to eight cycles of sequential anthracyclin/taxane-based chemotherapy in combination with trastuzumab and pertuzumab are considered as standard-of-care; of note, no relevant increase in cardiac toxicity rates was observed with short-term concurrent administration of anthracyclines and HER2-targeted therapy [24] but caution is mandated in patients with pre-existing cardiac risk factors.

While the addition of the first-generation HER2 tyrosine kinase inhibitor (TKI) lapatinib to trastuzumab and chemotherapy increased pCR rates as well, this approach was deemed less successful due to the increased toxicity when combining lapatinib and chemotherapy (NeoALTTO) [26].

Trials of bevacizumab, a humanized monoclonal antibody targeting VEGF-A, to neoadjuvant chemotherapy in HER2-negative breast cancer yielded contradicting results [13, 27]. In addition, a dissociation of pCR and long-term outcome was suggested in patients receiving bevacizumab in the ARTemis trial [28]; this phenomenon may be explained by the lack of activity of bevacizumab against micrometastases suggesting that bevacizumab should not be used as a component of neoadjuvant treatment.

Interesting novel approaches include the addition of denosumab, a monoclonal antibody targeting the RANK-ligand RANKL, to neoadjuvant chemotherapy in unselected breast cancer patients as RANKL apparently plays a key role in breast carcinogenesis (GeparX; EudraCT No.: 2015-001755-72) [29]. Furthermore, the randomized phase II trial GeparOLA compares paclitaxel combined with the PARP-inhibitor olaparib with paclitaxel/carboplatin (both followed by epirubicin/cyclophosphamide; EC) in patients harbouring germline and/or tumour *BRCA* mutations and/or a high homologous recombination deficiency (HRD) score (GeparOLA; EudraCT No.: 2015-003509-41). This study will therefore help in defining the role of carboplatin in *BRCA* mutation carriers as well as the safety and efficacy of olaparib in the neoadjuvant setting in different patient populations.

### Immune checkpoint inhibitors

Immune checkpoint modulators are a class of drugs counteracting tumour-associated inhibition of T‑lymphocyte activation. These antibodies targeting PD-1, PD-L1 or CTLA-4 are highly active and well established in the treatment of melanoma or non-small cell lung cancer while development in breast cancer was delayed. Recent clinical research focused on TNBC and early results suggested that a combination of chemotherapy with checkpoint inhibitors may improve the activity of this treatment approach in breast cancer [30].

In the neoadjuvant setting, a trial from the phase II I‑SPY2 platform suggested that the addition of pembrolizumab, a monoclonal antibody targeting PD-1, to standard neoadjuvant chemotherapy increases pCR rates in early stage breast cancer [31]. A total number of 69 patients with TNBC or high-risk luminal breast cancer were randomized to paclitaxel plus pembrolizumab followed by AC; 180 patients served as control group. Results of I‑SPY2 trials are presented as estimates, as these data predict the expected outcome of the therapies und investigation in a phase III trial. In the overall population, estimated pCR rates were 46% (95% CI 0.34–0.58) with pembrolizumab *versus* 16% (95% CI 0.06–0.27) without, resulting in a predictive probability of success in a phase III trial of 99%. As expected, this difference was most pronounced in TNBC (estimated pCR rate 60% *vs*. 20%) but a benefit was observed in high-risk luminal patients as well (estimated pCR rate 34% *vs*. 13%) for a predictive probability of success of 88%.

Furthermore, preliminary data from the GeparNuevo trial (EudraCT No.: 2015-002714-72) high-lighted the safety of the PD-L1 directed antibody durvalumab when combined with preoperative chemotherapy consisting of nab-paclitaxel and EC [32] and indicated a nonsignificant pCR increase (44.2% vs. 53.4%; OR 1.53; *p* = 0.182) [33]. Finally, the phase Ib KEYnote-173 study added pembrolizumab to neoadjuvant treatment consisting of weekly nab-paclitaxel × 12 (with or without carboplatin AUC6 once every three weeks) followed by four cycles of once every three weeks AC in 20 TNBC patients [34]. In the nab-paclitaxel cohort, the pCR rate in breast and axilla was 50% (90% CI 22–78%) and 90% in patients receiving carboplatin plus nab-paclitaxel (90% CI 61–100%); as expected, toxicity was higher in the carboplatin group. Of note, no dose-limiting toxicity linked to pembrolizumab was observed.

Currently, several phase III trials incorporating PD-1 or PD-L1 inhibitors such as the joint NSABP/GBG GeparDouze (NCT03281954) study are ongoing or about to be started; provided positive results of these studies are obtained, immune checkpoint inhibitors may become an important component of TNBC therapy.

In luminal breast cancer, the French Unicancer Group tests different strategies in order to increase the immune reactivity of luminal breast cancer in the phase II ULTIMATE trial (NCT02997995). Patients eligible for neoadjuvant endocrine therapy will receive different immune-attractants (e. g. tremelimumab, an antibody targeting CTLA-4) plus the steroidal aromatase-inhibitor exemestane and patients presenting with >10% CD8+ cells in the tumour after 3 weeks will then continue with exemestane plus durvalumab for another six months. This strategy may prove important for extending the concept of immunotherapy beyond TNBC.

## Summary

Neoadjuvant chemotherapy has become a potential standard treatment approach and is regarded as the preferred treatment strategy in HER2-positive and triple-negative early stage breast cancer. As pCR predicts for improved long-term outcome, clinical trials commonly focus on increasing pCR rates. With the marked exception of carboplatin, adding further chemotherapeutic agents has been of limited success; in contrast, addition of anti-HER2 targeted agents has vastly improved pCR rates in HER2-positive disease and may even allow for chemotherapy de-escalation. Finally, promising novel strategies, among them immune checkpoint inhibitors, are currently under clinical investigation.
